# The effects of Tiszasüly and Kolop mud pack therapy on knee osteoarthritis: a double-blind, randomised, non-inferiority controlled study

**DOI:** 10.1007/s00484-019-01764-4

**Published:** 2019-08-03

**Authors:** Márta Király, Eszter Kővári, Katalin Hodosi, Péter V. Bálint, Tamás Bender

**Affiliations:** 1 Petz Aladár County Teaching Hospital, Híd u.2., Győr, H-9025 Hungary; 2 Doctoral School of Clinical Medicine, University of Szeged, Korányi fasor 6., Szeged, H-6720 Hungary; 3 School of PhD Studies, Semmelweis University, Üllői út 26., Budapest, H-1085 Hungary; 4 Medical and Health Science Center, University of Debrecen, Nagyerdei körút 98., Debrecen, H-4012 Hungary; 5 3rd Rheumatology Department, National Institute of Rheumatology and Physiotherapy, Frankel L. u. 25-29., Budapest, H-1023 Hungary; 6 Polyclinic of Hospitaller Brothers of St. John of God, Árpád fejedelem útja 7., Budapest, H-1023 Hungary; 7 Department of Orthopedics, Faculty of Medicine, Doctoral School of Clinical Medicine, University of Szeged, Szeged, Hungary

**Keywords:** Mud pack therapy, Osteoarthritis of the knee, Hot-pack therapy, Non-inferiority study

## Abstract

The aim of this non-inferiority study was to evaluate and compare the effects of Tiszasüly and Kolop mud pack therapy on pain, function and quality of life in patients with knee osteoarthritis. In this double-blind, randomised, follow-up study, 60 patients with knee osteoarthritis were treated with either Tiszasüly hot mud pack (group 1) or with Kolop hot mud pack (group 2) on 10 occasions for 2 weeks (10 working days). One hundred millimetre visual analogue scale (VAS) for knee pain, the Western Ontario and McMaster Universities Arthritis Index (WOMAC), the Knee injury and Osteoarthritis Outcome Score (KOOS), the Lequesne Index for physical function and EuroQoL-5D for quality-of-life measurements were recorded at baseline, at the end of treatment (week 2) and 3 months later (week 12). In both groups, all measured parameters improved significantly from the baseline until the end of treatment and during the follow-up period (*p* < 0.05). There were no significant differences between the groups in terms of the WOMAC, KOOS, EQ-5D and Lequesne Index at any visits. Knee pain improved in both groups at week 2 and week 12; the only significant difference visible between the groups was at the end of the treatment in favour of the Tiszasüly mud pack group (*p* = 0.009). Tiszasüly and Kolop mud packs both have a favourable effect on knee pain, physical function and quality of life in patients with knee osteoarthritis. Our results proved non-inferiority of Tiszasüly mud pack.

## Introduction

Osteoarthritis (OA) is the most prevalent musculoskeletal disease, which burdens not only the patients but also the society. Etiology of this multifactorial disease is still unknown, that is why therapeutical strategies for ease of pain are limited. Pathophysiologic processes lead to anatomical damage and functional insufficiency of the joint that may cause limitation of self-care and quality of life (Helmick et al. 2008; Lawrence et al. 2008; Bijlsma et al. 2011). Various factors may play a role in the development of OA like age, sex, occupation, weight, recreation, and diet, but also genetic and environmental factors and mechanical stress are thought to be in the background of the disease. Based on European surveys, in treating degenerative musculoskeletal diseases, we can intensely count not only on direct (medical treatment), but also on indirect costs (e.g. working disability, expenses due to disabled self-care) (Rabenda et al. 2006).

Balneotherapy deals with the effects and medical use of natural mineral waters, gases and peloids on preventive, therapeutic and rehabilitative purposes (Gutenbrunner et al. 2010). Pelotherapy is a considerable part of balneotherapy, which plays an important role in the local treatment of knee osteoarthritis (Meng and Huang 2018). Peloids are muddy suspensions with healing properties (Gomes et al. 2013). International nomenclature and classification of mud or peloid are still not uniform. Peloids are a mixture of fine-grained materials of natural (geologic and/or biologic) origins, mineral water or sea water, and commonly organic compounds from a biological metabolic activity. Maturation could take place either in natural or in artificial (e.g. in a tank) environments. During maturation, the growth of microorganisms originates several metabolic products. Medical muds/peloids have a high heat retention and low heat conduction capacity; therefore, they can provoke endogen heat formation (cooling time is rather long; they do not cause skin burn). Some peloids may also contain estrogen, which could be responsible for their analgesic effect. In Hungary, 6 types of medical peloids are used: peloids from Makó, Kolop, Héviz, Hajdúszoboszló, Alsópáhok (Georgikon), and peloid from Austria, Neydharting, which is a shallow peat. These peloids can be classified in three groups based on their origin: (1) the Makó, Kolop and Hajdúszoboszló peloids are inorganic, because of their notable mineral content, and very little organic component; (2) the Héviz peloid represents the mixed peloids, which are rich in volcanic minerals and contain 20–25% Sphagnum peat; and (3) the Georgikon’ peat belongs to the organic peloids containing a large amount of organic components and humins. Mud/peloid therapy can be used as an active or passive treatment. In case of an active therapy, patients are able to move in the mud (mud bath, mud lake), whereas passive treatment means topical use like mud pack or mud wrap.

Kolop peloid has been used in most of the spas in Budapest since the early twentieth century. Kolop is located in Jász-Nagykun-Szolnok county (Hungary) under the municipality of Tiszasüly. Production of Tiszasüly and Kolop peloid is next to each other, thus their composition is similar; it is within a natural fluctuation (Table 1). In our non-inferiority study, we postulated that the clinical effectiveness of the two very similar peloids are alike. As judgement of medical utilisation of mineral waters or peloids is very strict in Hungary, we needed to conduct this clinical study to gain the curative qualification of Tiszasüly peloid.

**Table 1:** The chemical composition of the Kolop and Tiszasüly mud

Mud	Kolop	Tiszasüly		Kolop	Tiszasüly
SiO_2_	60.05%	69.1%	CaO	1.54%	2.53%
TiO_2_	0.54%	–	MgO	2.10%	–
Al_2_O_3_	17.91%	17.58%	Na_2_O	0.89%	–
Fe_2_O_3_	4.34%	–	K_2_O	2.39%	2.73%
FeO	2.38%	–	CO_2_	0.29%	–
MnO	0.05%	–	Cl^−^	0.05%	–
P_2_O_5_	0.14%	–	SO_3_	0.28%	–
Organic content	1.53%	1.475%			

Physical properties of Kolop peloid, such as particle size, rheological properties, its radium content, and heat storage capacity makes it appropriate for balneotherapy, in particular for musculoskeletal disorders. The particle size and distribution of the peloid is more than 90% in the ideal range (0.02–0.002 mm). Its radium (^226^Ra) content (4.18 mg radium/10 tons) may have an important role in therapy. The chemical composition is summarised in Table 1. Hungarian experimental studies performed by comet assay on Eisenia coelomocytes have ruled out the potential genotoxic effects of Kolop peloid and proved that it inhibits the reproductive capacity of Eisenia and also root elongation (Gerencsér et al. 2015; Varga 2012).

### Aim of the study

The aim of our non-inferiority study was to evaluate and compare the effects of Tiszasüly and Kolop mud pack therapy on pain, function and quality of life in patients with knee osteoarthritis.

## Patients and methods

Our study protocol met the principles of the Helsinki declaration. Study participants were informed verbally about the protocol, received written information and they signed the Informed Consent Form before the initiation of the study. This randomised, controlled, assessor-blinded trial was approved by the Regional Research Ethics Committee, Petz Aladár County Teaching Hospital (approval number: 76-1-9/2016) and registered in ClinicalTrials.gov (NCT03826511). Participants were recruited from patients of the Department of Rheumatology and Physiotherapy of Petz Aladár County Teaching Hospital. The study was conducted between August 2016 and February 2018.

### Inclusion criteria

We enrolled patients over 40 years of age, who are capable to answer questionnaires and have clinically and radiologically bilateral knee osteoarthritis according to EULAR recommendation (mechanical knee pain, morning stiffness < 30 min, reduced knee function, radiological signs: Kellgren-Laurence radiological grade 2–3; grade 2, osteophyte formation and possible joint space narrowing; grade 3, multiple osteophytes and definite joint space narrowing, sclerosis and possible bone deformity) (Zhang et al. 2009). Patients must have had initial spontaneous knee pain ≥ 50 mm on Visual Analogue Scale.

### Exclusion criteria

Exclusion criteria were infection, fever, ongoing malignant tumour, neuropathy of the lower extremities, skin changes of the treated area, high blood pressure, progrediating heart failure (NYHA Class II–IV), inflammatory rheumatic disease, prior arthroplasty of the knee, intraarticular steroid or viscosupplementation therapy within 3 months prior treatment, physiotherapy of the knee within 3 months prior treatment, and inflammatory knee osteoarthritis.

### Randomisation

A concealed allocation random assignment of the enrolled patients to the treatment groups was performed by an independent study person (using Microsoft Excel software) who did not meet any of the patients and did not participate in the course of the study either.

### Blinding method

Neither the testing investigators and assistants nor the patients were aware of the treatment assignments both at the start and the end of the study. The statistician was not involved in the randomisation process either.

### Recruitment of the patients

**Fig. 1:**
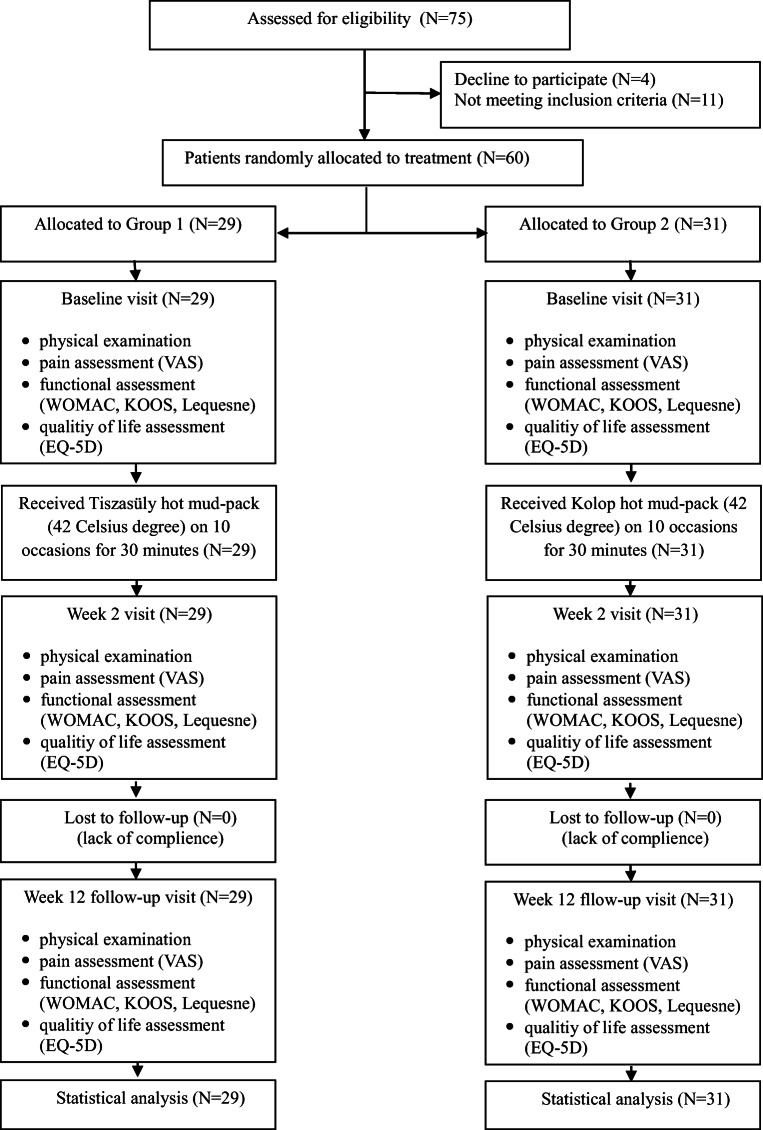
The disposition of patients

Altogether, 75 patients were included and 60 patients were randomised. Eleven patients did not meet inclusion criteria, and four patients revoked their consent. Following randomisation, 60 patients were grouped into 2 arms: the group 1 and group 2. The allocation and the type of mud pack in the groups were concealed by using sealed, opaque envelopes. Twenty-nine patients out of 60 (mean age, 65.03 ± 8.56 years; male/female, 10/19) were assigned to group 1 and 31 patients (mean age, 66.67 ± 7.62 years; male/female, 8/23) to the Group 2 (Fig. 1).

There were no significant differences among the two groups in gender proportions, comorbidities, knee osteoarthritis duration, and radiological score distribution (Table 2). The most frequent co-morbidities in both treatment groups were cardiovascular diseases. About one-third of the patients had metabolic diseases like hyperlipidaemia and endocrine diseases like diabetes. The mean duration of the knee osteoarthritis was 5–6 years. The majority of the patients had grade 2 Kellgren-Laurence radiological score. Approximately two-thirds of the patients did not have osteoarthritis besides the knees, and one-third had hip OA.

**Table 2:** Demographic characteristics of patients

	Group 1 (*n* = 29)	Group 2 (*n* = 31)	*p*
Age (years)	65.03 ± 8.56	66.67 ± 7.62	0.435
Male/Female	10/19	8/23	0.464
Comorbidities:			
Cardiovascular diseases	26 (89.6%)	28 (90.3%)	1.000
Endocrine diseases	8 (27.6%)	7 (22.6%)	0.881
Metabolic diseases	8 (27.6%)	13 (41.9%)	0.372
Gastrointestinal diseases	5 (17.2%)	3 (9.6%)	0.465
Benign prostate hyperplasia	3 (10.3%)	2 (6.4%)	0.666
Psychiatric diseases	3 (10.3%)	2 (6.4%)	0.666
Osteoporosis	4 (13.8%)	6 (19.3%)	0.732
Knee OA duration (years)	6.57 ± 5.30	4.88 ± 3.53	0.353
Radiological score distribution			
Kellgren-Laurence grade 2	19 (65.5%)	25 (80.6%)	0.302
Kellgren-Laurence grade 3	10 (34.5%)	6 (19.3%)	
Other OA			
Hip OA	11 (37.9%)	11 (35.5%)	0.844
Shoulder OA	2 (6.9%)	4 (12.9%)	0.672
Ankle OA	2 (6.9%)	1 (3.2%)	0.606

### Interventions

Group 1 received Tiszasüly hot mud pack (42 °C), group 2 received Kolop hot mud pack (42 °C) on the painful knee once a day for 30 min on 10 occasions (2 weeks). The two mud packs had similar package and physical properties. The treatment was performed by an independent, blinded, qualified assistant. Patients were lying during the therapy and after 30 min the mud-pack was washed off by the assistant. The applied mud was discarded at the end of the treatment.

### Outcome parameters

At the inclusion, patients’ ages and genders were recorded. During each visit, we examined the patient to assess range of motion, tenderness or swelling. We also applied a 100-mm visual analogue scale (VAS) to assess rest/spontaneous pain level. Functional impairment was measured by 3 different questionnaires: (1) the Western Ontario and McMaster Universities Arthritis Index (WOMAC) has 24 questions to evaluate pain (5 questions), physical function (17 questions) and stiffness (2 questions). (2) The Knee injury and Osteoarthritis Outcome Score (KOOS) is a self-administered instrument that was developed as an extension of the WOMAC Osteoarthritis Index. It can be used for short-term and long-term follow-up of knee osteoarthritis. The KOOS is composed of five separately scored subscales: pain, various symptoms, activities of daily living (ADL), function in sport and knee-related quality of life (QOL) (Roos and Lohmander 2003). (3) Lequesne Algofunctional Index has 10 questions; it has five questions pertaining to pain or discomfort, 1 question about maximum walking distance, and 4 questions about function in daily living. The total score is between 0 and 24. Lower scores mean less functional impairment. Quality of life was measured by EuroQoL-5D (EQ-5D) questionnaire. It has five dimensions (mobility, self-care, usual activities, pain/discomfort, anxiety/depression), where patients are asked to rate their health problems on 5 levels (no problems, slight, moderate, severe or extreme problems). It also contains a self-rating of health status using a visual analogue scale (EQ VAS) ranging from 0 to 100 (where 0 means the worst and 100 means the best health status). EQ-5D was valued based on a standardised time trade-off (TTO) for the general population in the United Kingdom (UK).

Outcome parameters were recorded by a blinded rheumatologist before the start of the therapy (week 0), immediately after the therapy series (week 2) and 3 months later (week 12).

### Statistical analysis

The statistical analysis was processed by the IBM SPSS 25 software. Data distribution was investigated with the Kolmogorov–Smirnov test. We found a non-normal distribution; the data were calculated by the Mann–Whitney and Wilcoxon test and are represented as the mean ± SD. The measurements of differences between groups were carried out by the Mann–Whitney test. We handled missing data using the last observation carried forward (LOCF) method. *p* values < 0.05 were considered significant. We did not use an intention to treat analysis approach. The power analysis done by the G power 3.1.9.2 programme was calculated from VAS pain values at week 2 using the Mann–Whitney non-parametric test. The power proved to be 84% in case of 29 and 31 sample sizes.

## Results

Both groups demonstrated similar changes during the study in all parameters.

Spontaneous pain significantly decreased in both groups after therapy and at 12 weeks follow-up (*p*0–1 < 0.001, *p*0–2 < 0.001); however, the difference between week 2 and week 12 was not significant. Immediately after therapy, the Tiszasüly mud pack group (group 1) showed better improvement (*p* = 0.009) compared with group 2 (Kolop mud pack).

Knee function impairment significantly improved in both the Tiszasüly and the Kolop mud pack groups for the week 2 and week 12 visits measured by WOMAC and the Lequesne index (group 1, *p*0–1 = 0.002, *p*0–2 = 0.001, and *p*0–1 = 0.001, *p*0–2 = 0.001 respectively; group 2, *p*0–1 < 0.001, *p*0–2 < 0.001, and *p*0–1 < 0.001, *p*0–2 = 0.004 respectively). The KOOS score showed decreasing impairment in both groups, but significant changes were demonstrated only in the Kolop mud pack group (group 2, *p*0–1 = 0.046, *p*0–2 = 0.039; group 1, *p*0–1 = 0.991, *p*0–2 = 0.905).

As to quality of life of patients measured by EuroQoL-5D, we found significant improvement in both groups (group 1, *p*0–1 = 0.039, *p*0–2 = 0.028; group 2, *p*0–1 < 0.001, *p*0–2 < 0.001), there were no significant differences between the groups at each visit. EQ-5D VAS score increased so in group 1 as in group 2, and the changes were significant in both groups (group 1, *p*0–1 = 0.024, *p*0–2 = 0.011; group 2, *p*0–1 < 0.001, *p*0–2 < 0.001) (Table 3).

**Table 3:** Means ± SD at baseline, at end of week 2 and week 12 for the two study groups (Group 1, patients receiving Tiszasüly hot mud pack; and group 2, patients receiving Kolop hot mud pack) and between-group differences at end of week 2 and week 12 show the effects of Tiszasüly and Kolop mud packs on pain, function, and quality of life in patients with knee osteoarthritis

Measured clinical variables	Treatment groups	Baseline visit		Week 2 visit		Week 12 visit		*p* 1–2 (significance level, difference between visits)	*p* 1–3 (significance level, difference between visits)	*p* 2–3 (significance level, difference between visits)
		Mean ± SD	*p* (significance level, between group difference)	Mean ± SD	*p* (significance level, between group difference)	Mean ± SD	*p* (significance level, between group difference)			
WOMAC sum.	Tiszasüly (*n* = 29)	888.62 ± 354.47	0.188	651.10 ± 382.90	0.126	608.17 ± 445.19	0.201	**0**.**002**	**0**.**001**	0.399
	Kolop (*n* = 31)	1048.74 ± 405.61		808.77 ± 389.09		773.35 ± 491.16		**< 0**.**001**	**< 0**.**001**	0.445
Koos score	Tiszasüly (*n* = 29)	52.15 ± 9.46	0.083	50.98 ± 21.68	0.790	50.57 ± 21.63	0.717	0.991	0.905	0.762
	Kolop (*n* = 31)	57.40 ± 12.81		51.68 ± 17.34		49.81 ± 19.69		**0**.**046**	**0**.**039**	0.943
EQ5D score	Tiszasüly (*n* = 29)	0.613 ± 0.177	0.488	0.694 ± 0.249	0.893	0.704 ± 0.265	0.327	**0**.**039**	**0**.**028**	0.689
	Kolop (*n* = 31)	0.490 ± 0.307		0.734 ± 0.146		0.652 ± 0.255		**< 0**.**001**	**< 0**.**001**	0.193
EQ5D VAS score	Tiszasüly (*n* = 29)	0.601 ± 0.145	0.577	0.680 ± 0.199	0.911	0.700 ± 0.213	0.313	**0**.**024**	**0**.**011**	0.647
	Kolop (*n* = 31)	0.534 ± 0.192		0.708 ± 0.150		0.655 ± 0.187		**< 0**.**001**	**< 0**.**001**	0.214
VAS score of knee pain	Tiszasüly (*n* = 29)	57.83 ± 14.16	0.329	24.10 ± 21.93	**0**.**009**	29.52 ± 26.15	0.296	**< 0**.**001**	**< 0**.**001**	0.160
	Kolop (*n* = 31)	61.39 ± 14.56		36.61 ± 17.50		35.16 ± 25.02		**< 0**.**001**	**< 0**.**001**	0.509
Lequesne sum index	Tiszasüly (*n* = 29)	10.45 ± 2.65	0.784	8.21 ± 3.93	0.953	7.72 ± 4.17	0.267	**0**.**001**	**0**.**001**	0.354
	Kolop (*n* = 31)	10.95 ± 3.70		8.00 ± 3.68		9.24 ± 4.49		**< 0**.**001**	**0**.**004**	0.046

No adverse events were noted or recorded during this study.

## Discussion

Balneotherapy is a conventional treatment of osteoarthritis (Forestier et al. 2016; Kulisch et al. 2014; Fioravanti et al. 2017; Karagülle et al. 2007). Based on best-available evidence, the new OARSI (Osteoarthritis Research Society International) guideline, updated in 2014, recommends balneotherapy besides intra-articular corticosteroids and oral non-steroidal anti-inflammatory drugs (NSAIDs) for the treatment of multiple-joint osteoarthritis with relevant co-morbidities (McAlindon et al. 2014). Peloids have been used for the treatment of musculoskeletal diseases for a long time and several studies have confirmed their effectiveness in osteoarthritis. In 2008, Turkish authors compared direct mud pack and nylon-covered mud pack on knee OA and revealed a better outcome in the directly applied mud group (Odabasi et al. 2008). Similar results are published by Hungarian authors investigating the effects of Héviz mud on patients with hand osteoarthritis. The treatment group received mud applied directly to both hands, whereas the control group received mud to both hands with a nylon layer that separated the skin from the mud. Both groups showed improvement at the end of treatment and after 16 weeks. However, the patients directly treated with mud, showed a significantly better improvement in some VAS scale parameters compared with the control group (Gyarmati et al. 2017). A quantitative meta-analysis of 7 studies (410 patients) in 2013 also confirmed the favourable effect of mud therapy on pain relief in patients with knee OA (Liu et al. 2013). In our study, we confirmed that the clinical effects of the 2 muds (Tiszasüly and Kolop) are basically the same, there was no significant difference between them, though Tiszasüly mud-pack showed better improvement in one parameter right after treatment. This corresponds to the fact, that production of the 2 muds is located very close to each other and the physical and chemical parameters of both muds are the same (Table 1). In a randomised, controlled, follow-up study, Hungarian authors evaluated the effects of Kolop peloid as part of combined physio- and balneotherapy treatment on knee osteoarthritis in the day hospital care setting. Peloid therapy combined with mineral water bathing, aquatic exercise and magnetotherapy significantly improved pain, function and quality of life compared with physio- and balneotherapy without peloid therapy (Horváth et al. 2013).

In another Hungarian double-blind RCT, the effects of Neydharting mud pack therapy were evaluated compared with hot-pack with similar physical properties (viscosity, plasticity, adherence to skin, water-binding capacity and colour) to that of the Neydharting mud. The clinical outcome parameters improved in both groups, which can be explained by the similar physical properties. There were no significant differences between the 2 groups, but the improvement in the treated group was greater than in the control group. The need for analgesics and NSAIDs decreased in the control group, while a significant change was observed in the mud-treated group by the follow-up visit. This might indicate a special chemical effect of the mud (Tefner et al. 2013). A recent meta-analysis verified the suspected effect of chemical components in balneotherapy (Morer et al. 2017). Based on the results of an Italian study, mud bath therapy can decrease the serum level of adiponectin and resistin that may play a protective role in the course of knee osteoarthritis (Fioravanti et al. 2015b). As to chondroprotective effects of mud therapy, it was demonstrated in 2 different studies, that mud compress reduces the urine levels of C telopeptide fragment of collagen type II (uCTX-II) and increases the serum levels of C-terminal crosslinked telopeptide type II collagen (CTX-II), perhaps due to an increase in cartilage turnover induced by thermal stress (Gungen et al. 2016; Pascarelli et al. 2016). In an experimental study, Hungarian authors investigated the anti-inflammatory and analgesic effects of Héviz thermal water and mud in monosodium iodoacetate-induced osteoarthritis and Complete Freund’s adjuvant-induced rheumatoid arthritis murine models. The treatment group received Héviz thermal water and mud pack, the control group received tap water and sand. Balneotherapy did not influence mechanical hyperalgesia, weight bearing, or oedema formation in the rheumatoid arthritis models, but had antinociceptive and anti-inflammatory effects in osteoarthritis (Tékus et al. 2018).

Fioravanti et al. published notable data about the long-term (12 months) effect of mud bath therapy added to usual treatment in patients with knee osteoarthritis (Fioravanti et al. 2015a). Besides the clinical effects, cost effectiveness of mud therapy is also important (Ciani et al. 2017). In the recent meta-analysis of 12 RCTs, spa therapy and mud therapy are discussed together, and found to be effective in the treatment and in the secondary prevention of knee OA (Fraioli et al. 2018).

All in all, based on our study and literature data, we can conclude, that mud therapy has been proved to be effective and safe in the treatment of knee osteoarthritis. It did not have any side effects in our patients with co-morbidities. It could be a good therapeutic choice not only in early osteoarthritis, but after several years disease duration. Despite the increasing evidence of the favourable effects of balneotherapy and mud therapy, they are traditionally used mainly in countries rich in thermal waters. This fact can interfere the appearance of mud therapy in guidelines of non-pharmacological treatment of osteoarthritis, although there are several excellent, well-designed studies based on consort statement available.

### Limitation of the study

Increasing the number of patients would power our study, though this number was enough to draw conclusions. We are planning to extend the follow-up period to 6 and 9 months.

## Conclusion

Based on our double-blind, controlled pilot study, we can conclude, that both Tiszasüly and Kolop mud packs have a favourable effect on knee pain, physical function and quality of life in patients with knee osteoarthritis. We could not find any significant difference in the clinical effects of the 2 muds, so our results proved the non-inferiority of Tiszasüly mud pack.
